# Persistence and Protective Potential of SARS-CoV-2 Antibody Levels After COVID-19 Vaccination in a West Virginia Nursing Home Cohort

**DOI:** 10.1001/jamanetworkopen.2022.31334

**Published:** 2022-09-13

**Authors:** Katy Smoot, Jianbo Yang, Danyel Hermes Tacker, Shelley Welch, Maryam Khodaverdi, Wes Kimble, Sijin Wen, Ayne Amjad, Clay Marsh, Peter L. Perrotta, Sally Hodder

**Affiliations:** 1 Department of Pathology, Anatomy, and Laboratory Medicine, West Virginia University School of Medicine, Morgantown; 2 Clinical & Translational Sciences Institute, West Virginia University, Morgantown; 3 Department of Epidemiology and Biostatistics, West Virginia University, Morgantown; 4 West Virginia Department of Health and Human Resources, Charleston; 5 Department of Medicine, West Virginia University, Morgantown

## Abstract

**Importance:**

West Virginia prioritized SARS-CoV-2 vaccine delivery to nursing home facilities because of increased risk of severe illness in elderly populations. However, the persistence and protective role of antibody levels remain unclear.

**Objective:**

To examine the persistence of humoral immunity after COVID-19 vaccination and the association of SARS-CoV-2 antibody levels and subsequent infection among nursing home residents and staff.

**Design, Setting, and Participants:**

In this cross-sectional study, blood samples were procured between September 13 and November 30, 2021, from vaccinated residents and staff at participating nursing home facilities in the state of West Virginia for measurement of SARS-CoV-2 antibody (anti–receptor binding domain [RBD] IgG). SARS-CoV-2 infection and vaccination history were documented during specimen collection and through query of the state SARS-CoV-2 surveillance system through January 16, 2022.

**Exposure:**

SARS-CoV-2 vaccination (with BNT162b2, messenger RNA-1273, or Ad26.COV2.S).

**Main Outcomes and Measures:**

Anti-RBD IgG levels were assessed using multivariate analysis to examine associations between time since vaccination or infection, age, sex, booster doses, and vaccine type. Antibody levels from participants who became infected after specimen collection were compared with those without infection to correlate antibody levels with subsequent infection.

**Results:**

Among 2139 SARS-CoV-2 vaccinated residents and staff from participating West Virginia nursing facilities (median [range] age, 67 [18-103] years; 1660 [78%] female; 2045 [96%] White), anti-RBD IgG antibody levels decreased with time after vaccination or infection (mean [SE] estimated coefficient, −0.025 [0.0015]; *P* < .001). Multivariate regression modeling of participants with (n = 608) and without (n = 1223) a known history of SARS-CoV-2 infection demonstrated significantly higher mean (SE) antibody indexes with a third (booster) vaccination (with infection: 11.250 [1.2260]; *P* < .001; without infection: 8.056 [0.5333]; *P* < .001). Antibody levels (calculated by dividing the sample signal by the mean calibrator signal) were significantly lower among participants who later experienced breakthrough infection during the Delta surge (median, 2.3; 95% CI, 1.8-2.9) compared with those without breakthrough infection (median, 5.8; 95% CI, 5.5-6.1) (*P* = .002); however, no difference in absorbance indexes was observed in participants with breakthrough infections occurring after specimen collection (median, 5.9; 95% CI, 3.7-11.1) compared with those without breakthrough infection during the Omicron surge (median, 5.8; 95% CI, 5.6-6.2) (*P* = .70).

**Conclusions and Relevance:**

In this cross-sectional study, anti-RBD IgG levels decreased after vaccination or infection. Higher antibody responses were found in individuals who received a third (booster) vaccination. Although lower antibody levels were associated with breakthrough infection during the Delta surge, no significant association was found between antibody level and infection observed during the Omicron surge. The findings of this cross-sectional study suggest that among nursing home residents, COVID-19 vaccine boosters are important and updated vaccines effective against emerging SARS-CoV-2 variants are needed.

## Key Points

**Question:**

What are the persistence and protective potential of SARS-CoV-2 antibody levels after vaccination in West Virginia nursing home residents and staff?

**Findings:**

In this cross-sectional study of 2139 participants from West Virginia nursing home facilities, antibody levels decreased with time after vaccination but were restored with booster doses. During the Delta surge, individuals experiencing breakthrough infection had significantly lower antibody levels, but no significant association was found between antibody level and infection observed during the Omicron surge.

**Meaning:**

Although these findings support the recommendation of booster doses to augment waning antibody responses, data are not conclusive in providing an antibody correlate of protection against infection.

## Teaser

This cross-sectional study of COVID-19 vaccinated residents and staff at West Virginia nursing home facilities examines SARS-CoV-2 spike antibody levels and the association with subsequent infection.

## Introduction

West Virginia prioritized COVID-19 vaccine delivery to nursing home residents because of reported higher rates of infection, severe illness, and death among older persons in chronic care facilities.^1,2^ In addition, nursing home staff were also prioritized for vaccination given their proximity to nursing home residents. As the COVID-19 pandemic unfolded, nursing home facilities have faced intense challenges combating outbreaks by implementing stringent safety protocols and offering increased testing and monitoring of nursing facility residents and staff.

The presence of antibodies (IgG) specific to the SARS-CoV-2 spike receptor binding domain (RBD) reflect previous vaccination or infection, whereas the presence of antibodies to nucleocapsid (N) protein reflect prior infection with SARS-CoV-2.^3^ The persistence of humoral immunity (as reflected by circulating antibody concentrations) after SARS-CoV-2 vaccination is still being characterized; however, studies^4,5^ suggest that anti–SARS-CoV-2 antibody levels may be lower in vaccinated patients residing in nursing homes and wane more rapidly. Because levels of spike IgG have been correlated with neutralizing antibody and accepted as a protective correlate for SARS-CoV-2 severe disease, recommendations for a third messenger RNA (mRNA) vaccination have been made, and a fourth vaccination was authorized for persons 50 years or older and others who are immunocompromised.^6,7^ In this study, we assessed SARS-CoV-2 spike antibody levels and their association with subsequent infection among West Virginia nursing home residents and staff who had previously received SARS-CoV-2 vaccination.

## Methods

### Study Design and Data Collection

Residents and staff from participating West Virginia nursing facilities who were 18 years or older and fully vaccinated with at least 2 doses of BNT162b2 (Pfizer) or mRNA-1273 (Moderna) or at least 1 dose of Ad26.COV2.S (Janssen/Johnson & Johnson) were eligible to participate in this cross-sectional study. Study involvement was voluntary, and data collection was limited to participating individuals. The exclusion criteria included the presence of fatally rapid disease likely to result in death within less than 6 months. Nursing home staff, residents, and residents’ families (if deemed appropriate by nursing home staff) were provided an information sheet explaining the study. A Health Insurance Portability and Accountability Act waiver of consent was approved by the West Virginia University Institutional Review Board. Data obtained were identifiable to the research team. Data were deidentified for the purposes of analysis. This study followed the Strengthening the Reporting of Observational Studies in Epidemiology (STROBE) reporting guideline.

A single serum specimen was collected from each participant between September 13 and November 30, 2021. At the time of sample collection, participants and/or nursing home staff provided the following information on participating individuals: name, date of birth, sex, race, nursing resident or staff, vaccine manufacturer and administration dates, and date of the last known COVID-19 infection. Specimens were collected as a 1-time blood draw in serum separator tubes with clot activator and transported to the West Virginia University Rapid Development Laboratory, a Clinical Laboratory Improvement Amendments–licensed laboratory, for SARS-CoV-2 IgG testing.

Any missing participant data and infection and vaccination data occurring after specimen collection, including documented SARS-CoV-2 infection, testing dates, SARS-CoV-2 polymerase chain reaction test results, vaccination dates, and SARS-CoV-2–related deaths, were obtained from the West Virginia Department of Health and Human Resources (WVDHHR). All participant WVDHHR records reported from the pandemic onset (WVDHHR SARS-CoV-2 records initiated on March 4, 2020) until the study end date of January 16, 2022, were accessed. To reduce the chance of missing data from typographic errors or abbreviations, fuzzy matching was implemented on patient information and reviewed for all possible matches within WVDHHR data set. In detail, we identified records having similar first name, last name, and date of birth and concluded records to be a match if 1 of following criteria were met: (1) having exact first name, last name, and month and day of birth; (2) having exact first name, last name, and year and month of birth; or (3) having exact first name, last name, and year and day of birth. We also concluded 2 records to be a match when the only difference between records was using an abbreviated first name instead of a full name, using a similar first or last name with 1 or 2 extra letters, or using a similar first or last name with 1 or 2 different letters.

In the context of this article, *fully vaccinated* refers to participants who completed initial vaccination series (2 doses of the BNT162b2 or mRNA-1273 vaccine or 1 dose of the Ad26.COV2.S vaccine). *Vaccine breakthrough infections* are SARS-CoV-2 infections that occur at least 14 days after full vaccination. *Boosted* is defined as participants who received a third dose of the BNT162b2 or mRNA-1273 vaccine.

### Serologic Assays

Serum was screened for anti-RBD and antinucleocapsid IgG with enzyme-linked immunosorbent assay (ELISA) using a modified protocol from Horspool et al.^8^ Briefly, a 1:125 dilution of sample was applied in duplicate to plates coated with recombinant RBD (2 μg/μL) or N (1 μg/μL) antigen followed by a 5-minute incubation. All incubations were performed at 30 °C with gentle shaking (1.5 Hz). After plate washing, secondary antibody buffer (1:1000 dilution of goat antihuman IgG secondary antibody [Invitrogen], horseradish peroxidase in 3% milk diluted in 1% phosphate-buffered saline, and 0.5% Tween-20) was applied to the wells, followed by a 9-minute incubation. After final plate wash, 3,3′,5,5′-tetramethylbenzidine– stabilized substrate for horseradish peroxidase (Promega) was aliquoted to wells and incubated for 3 minutes. The reaction was stopped using 3 mol/L hydrochloride. Absorbance was read at 450 nm using a Sunrise spectrophotometer (Tecan), and the index was calculated by dividing the sample signal by the mean calibrator signal. Calibrator material consisted of a 1:16 000 dilution of convalescent plasma. The World Health Organization (WHO) international reference panel for anti–SARS-CoV-2 immunoglobulin panel was obtained from the National Institute for Biological Standards and Control (NIBSC) (code 20/268), and the mean index of panel samples was calculated for comparison purposes.

Measurement of neutralizing antibody was assessed in a subset of 95 participants to evaluate correlation with anti-RBD IgG indexes; specimens were selected to account for the entire ELISA reportable range. Angiotensin-converting enzyme 2–binding inhibition assay (SARS-CoV-2 Panel 13 V-PLEX neutralization kit; Meso Scale Diagnostics) was performed according to the manufacturer’s instructions using a 1:10 dilution of participant serum. Electrochemiluminescence signal was obtained on a plate reader (QuickPlex SQ120 plate reader; Meso Scale Diagnostics). Data were analyzed by graphing linear regression of log electrochemiluminescence signal against corresponding IgG data obtained using ELISA.

### Statistical Analysis

Frequency distributions of various study sample characteristics were generated. The Wilcoxon rank sum test was used to assess the difference of continuous variables between subpopulations, whereas the Fisher exact test (or χ^2^ test in the case of vaccine type comparison) was used for categorical variables. Multivariate analysis was performed to evaluate the association between antibody indexes and independent variables, including time since vaccination, age, booster status, sex, and vaccine type. Antibody levels from participants who became infected after specimen collection were compared with those without infection to correlate antibody levels with subsequent infection. The linear model assessment was performed according to Akaike information criterion and statistical significance. A 2-sided *P* < .05 implies statistical significance in this study. Statistical calculations were performed using Prism-GraphPad software, version 9 (GraphPad Inc).

## Results

### Population Cohort and SARS-CoV-2 Infection History

Forty-one of West Virginia’s 95 nursing homes agreed to participate in the study. Reasons for nursing home nonparticipation included staffing issues, ongoing SARS-COV-2 institutional outbreaks that precluded external visitors, and inability of the hired phlebotomy organization to service the nursing home. Among those 41 nursing homes, there were 3109 residents and 2492 staff. The participating study population (ie, those who agreed to participate and met the inclusion criteria) included 2164 COVID-19 vaccinated participants, of whom 25 failed to provide sufficient samples for serologic testing and were excluded from the study. Among the 2139 participants included in the study (median [range] age, 67 [18-103] years; 1660 [78%] female; 2045 [96%] White, 44 [2%] Black, 7 [<1%] Asian, 1 [<1%] American Indian, 3 [<1%] native Hawaiian, and 39 participants [2%] who chose not to disclose their racial or ethnic status), 1086 were nursing home residents and 1053 were nursing home staff members (Table).

**Table.** Study Population Demographic Characteristics at the Time of Specimen Collection (September 13 to November 30, 2021)

Characteristic	No. (%)			*P* value
	Nursing home residents (n = 1086)	Nursing home staff (n = 1053)	Total (N = 2139)	
Age, median (range), y	81 (20-103)	49 (18-83)	67 (18-103)	<.001
Sex				
Male	348 (32)	131 (12)	479 (22)	<.001
Female	738 (68)	922 (88)	1660 (78)	
Race^a^				
American Indian	NA	NA	1 (<1)	<.001
Asian	NA	NA	7 (<1)	
Black	NA	NA	44 (2)	
Native Hawaiian	NA	NA	3 (<1)	
White	1065 (98)	980 (93)	2045 (96)	
Other^b^	5 (<1)	34 (2)	39 (2)	
Vaccine				
BNT162b2 (Pfizer)	616 (57)	587 (56)	1203 (56)	.09
mRNA-1273 (Moderna)	458 (42)	442 (42)	900 (42)	
Ad26.COV2.S (Janssen/Johnson & Johson)	12 (1)	20 (2)	32 (1)	
Unspecified or hybrid	0	4 (<1)	4 (<1)	
Vaccination status				
Fully vaccinated^c^				<.001
Not boosted	751 (69)	843 (80)	1594 (75)	
Boosted^d^	335 (31)	210 (20)	545 (25)	
Prior SARS-CoV-2 infection	372 (34)	236 (22)	608 (28)	<.001
Antibody results				
Anti-RBD IgG^e^				
Negative or equivocal (index <1.2)	96 (9)	41 (4)	137 (6)	<.001
Positive (index ≥1.2)	990 (91)	1012 (96)	2002 (94)	
Antinucleocapsid IgG index				
<1.7	632 (58)	814 (77)	1446 (68)	<.001
≥1.7	454 (42)	239 (23)	693 (32)	

Abbreviations: NA, not available; RBD, receptor binding domain.

^a^ A breakdown of race between nursing home residents and staff was not available for those whose race was reported as American Indian, Asian, Black, and Native Hawaiian.

^b^ Other indicates participants who chose not to disclose their racial or ethnic status.

^c^ Fully vaccinated is defined as receiving 2 doses of the BNT162b2 or mRNA-1273 or 1 dose of the Ad26.COV2.S vaccine.

^d^ Boosted is defined as receiving an additional (third) dose of BNT162b2 or mRNA-1273 vaccine.

^e^ Classifications were defined by internal validation testing.

**Figure 1.**
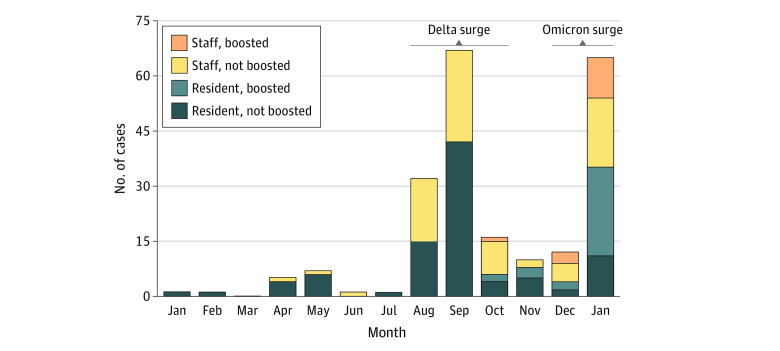
Distribution of Breakthrough SARS-CoV-2 Infections in West Virginia, January 2021 to January 2022 Boosted is defined as receipt of a third dose of the BNT162b2 or mRNA-1273 vaccine.

Compared with staff, the nursing home residents were older, more likely to be men, and more likely to have received a vaccine booster dose (335 [31%] compared with 210 [20%]; *P* < .001). At the time of specimen collection, a higher percentage of nursing home residents reported previous SARS-CoV-2 infections compared with staff (372 [34%] compared with 236 [22%]; *P* < .001) (Table). Most of these individuals reported infections that occurred before full vaccination status 478 [79%]) and between November 2020 and January 2021 (382 [63%]). Prior vaccine breakthrough infections were reported by 128 participants (6%) (71 residents [6%] and 57 staff [5%]) at sample receipt, of which 105 infections (82%) occurred between August and October 2021 during the SARS-CoV-2 Delta surge in West Virginia (data on file, West Virginia University). As stated in the Methods section, vaccine breakthrough infections were monitored after specimen collection using the WVDHHR COVID-19 surveillance system. On the basis of available data, a total of 219 participants (10%) within this cohort experienced vaccine breakthrough infections between January 22, 2021, and January 16, 2022, with 197 (90%) of these participants having documented infections between August 2021 and January 2022 during Delta and Omicron SARS-CoV-2 surges in West Virginia (Figure 1). Documented vaccine breakthrough infections were comparable between nursing home residents (119 [11%]) and staff (100 [9%]; *P* = .28). A total of 101 participants (including 51 residents) had documented breakthrough infections between October 2021 and January 2022, of whom 44 (44%) (25 residents [57%] and 13 staff [30%]) had received a booster dose before infection (based on West Virginia vaccination records). Notably, 65 infections occurred in January 2022, when virtually all identified variants in West Virginia were Omicron (data on file, West Virginia University). Of those 65 infections, 35 (54%) occurred among nursing home residents, 24 (69%) of whom had received a booster, compared with 30 infected nursing home staff, 11 (37%) of whom had received a booster (Figure 1). SARS-CoV-2 reinfections were documented in 16 individuals, with a median (range) of 388 (16-440) days between documented initial infection and reinfection. Of these 16 participants, 4 experienced both initial infection and reinfection after full vaccination.

### Association of Anti-RBD IgG Antibody Levels With Participant Demographic Characteristics

Anti-RBD IgG antibody indexes plotted against time since last exposure (ie, vaccination or infection) demonstrated an inverse association (mean [SE] estimated coefficient, −0.025 [0.0015]; *P* < .001) (eFigure 1 in the Supplement). Multivariate regression modeling of participants without a documented history of SARS-CoV-2 infection at the time of specimen collection and without serologic evidence of past infection (anti-N antibody indexes <1.7 [n = 1223]) demonstrated significantly lower antibody levels with older age (mean [SE] estimated coefficient, −0.032 [0.0044]; *P* < .001) and male sex (mean [SE] estimated coefficient, −0.679 [0.2147]; *P* = .002); higher antibody levels were associated with vaccination with the mRNA-1273 vaccine (mean [SE] estimated coefficient, 1.020 [0.1946]; *P* < .001) and receipt of a booster dose (mean [SE] estimated coefficient, 8.056 [0.5333]; *P* < .001). However, regression modeling for individuals with a known history of SARS-CoV-2 infection that occurred before specimen collection (n = 608) did not demonstrate significant associations with age (mean [SE] estimated coefficient, −0.004 [0.0104]; *P* = .72) or vaccine type (BNT162b2: mean [SE] estimated coefficient, 0.081 [0.4832]; *P* = .87; Ad26.COV2.S: mean [SE] estimated coefficient, 5.045 [3.561]; *P* = .16, compared with the mRNA-1273 vaccine) but demonstrated a significant association with sex (higher among men; mean [SE] estimated coefficient, 1.208 [0.4758]; *P* = .01) and booster receipt (mean [SE] estimated coefficient, 11.250 [1.2260]; *P* < .001). Of interest, a significantly higher percentage of nursing home residents had negative test results for anti-RBD IgG antibodies compared with staff (96 [9%] vs 41 [4%]; *P* < .001) (Table). A total of 308 participants (14%) (183 residents and 125 staff) with no reported history of infection at the time of specimen collection yielded anti-N indexes of 1.7 or higher, likely indicative of unknown or undocumented past infection.

### Assessment of Anti-RBD IgG Antibody Levels as a Correlate of Protection

Among the 95 participants identified with SARS-CoV-2 infection after specimen collection, 78 individuals had no record of a booster vaccine receipt after sample procurement. Among those 78 individuals (median [range] time after blood draw until infection: 71 [1-119] days), the median antibody index (calculated by dividing the sample signal by the mean calibrator signal) was 4.1 (95% CI, 2.9-6.8) compared with 5.8 (95% CI, 5.6-6.2) for individuals with no reported infection (*P* = .44).

**Figure 2.**
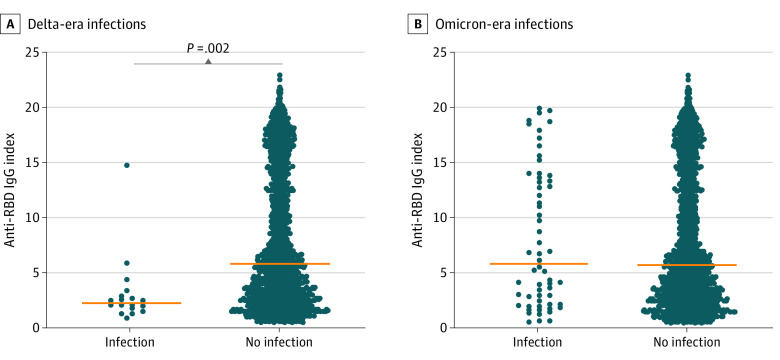
Comparison of Anti–Receptor Binding Domain (RBD) IgG Index Levels in Participants With Documented SARS-CoV-2 Infection Occurring After Sample Procurement and Those With No Evidence of Subsequent Infection Delta-era infections were infections that occurred between sample procurement and November 30, 2021. Omicron-era infections were infections that occurred between December 1, 2021, and January 16, 2022. Orange line represents the median index.

On the basis of available sequencing data (data on file, West Virginia University), these 78 infection cases were further subdivided into Delta-era infections (occurring after sample draw until November 30, 2021; n = 18) and Omicron-era infections (infections occurring between December 1, 2021, until study completion; n = 60). Significantly lower antibody indexes were observed in individuals with Delta-era infections (median, 2.3; 95% CI, 1.8-2.9) compared with individuals without infection (median, 5.8; 95% CI, 5.5-6.1) (*P* = .002) (Figure 2A). However, this trend was not observed in Omicron-era infections because there was no significant difference in antibody levels between individuals who were infected (median, 5.9; 95% CI, 3.7-11.1) and those who were not (median, 5.8; 95% CI, 5.6-6.2) (*P* = .70) (Figure 2B).

**Figure 3.**
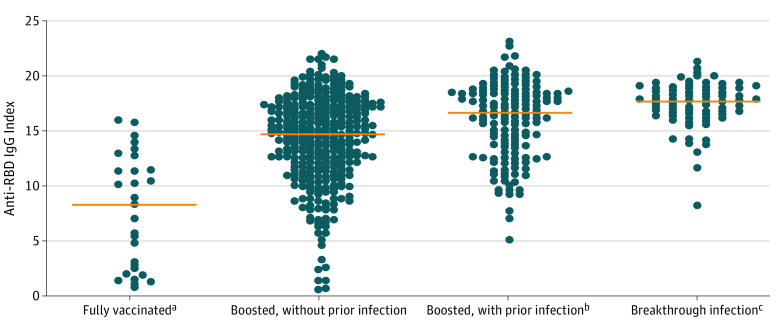
Distribution of Anti–Receptor Binding Domain (RBD) IgG Index Levels Among Various Participant Groups Data were from time-comparable cases with the most recent defined exposures (ie, vaccination or breakthrough infection) that occurred within 14 to 77 days before specimen collection. Individuals with breakthrough infections were fully vaccinated but not boosted. Orange line represents the median index. ^a^Significantly lower median antibody index compared with all other groups (all, *P* < .001). ^b^Significantly lower median antibody index compared with boosted individuals with previous infection (*P* < .001). ^c^Significantly higher median antibody index compared with all other groups (all, *P* < .001).

Figure 3 shows distribution of anti-RBD IgG index levels in participants with defined exposures (ie, vaccination or breakthrough infection). Data were analyzed from participants with exposures reported within 14 to 77 days before specimen collection. Fully vaccinated participants with no history of SARS-CoV-2 infection had significantly lower antibody indexes (median, 8.0; 95% CI, 2.5-11.3; n = 29) compared with (1) boosted individuals without previous infection (n = 339; median, 14.0; 95% CI, 13.1-14.7; *P* < .001), (2) boosted individuals with previous infection (n = 150; median, 16.6; 95% CI, 15.9-17.3; *P* < .001), and (3) nonboosted participants with breakthrough infection (n = 76; median, 17.7; 95% CI, 17.0-18.1; *P* < .001). Of these participant groups, significantly higher antibody levels (mean, 17.7) were observed for group 3 compared with the other 2 groups (mean, 16.6 and 14.0; *P* < .001).

As a robustness check, angiotensin-converting enzyme 2–binding inhibition assays, a surrogate for viral-based neutralization assays, were conducted on a subset of 95 participant samples (eFigure 2 in the Supplement). A strong correlation between neutralization potential and anti-RBD IgG indexes was demonstrated (*R*^2^ = 0.89). Corresponding mean indexes for the WHO SARS-CoV-2 IgG reference panel were also measured as a reference point for comparison and measured as follows: index of 1.3 (low WHO standard [NIBSC code 20/140] designated as 45 binding antibody units [BAU]/mL), 1.5 (low WHO standard [NIBSC code 20/144], 66 BAU/mL), 4.0 (middle WHO standard [NIBSC code 20/148], 205 BAU/mL), and 7.4 (high WHO standard [NIBSC code 10/150], 817 BAU/mL).

As of January 2022, 1 COVID-19–related death was reported within the cohort. This participant was a 76-year-old nursing home resident who was infected with SARS-CoV-2 at the time of specimen collection and died 9 days after serum collection. Anti-RBD and anti-N IgG indexes were negative (indexes were 0.5 for both tests) for this individual who had completed a 2-dose vaccination series approximately 7 months before specimen collection.

## Discussion

This cross-sectional study of SARS-CoV-2 antibody levels among nursing home residents and staff demonstrates anti-RBD IgG antibody levels decreased with time after vaccination or infection, consistent with other reports^9,10^ and supports recommendations for booster doses to augment immune responses. Fully vaccinated, nonboosted individuals who had previously experienced vaccine breakthrough infections had significantly higher antibody levels compared with boosted participants with or without a history of infection. This observation demonstrates that booster doses may not restore antibody levels equivalently to breakthrough infections. However, given that vaccination reportedly reduced the need for mechanical ventilation and death by 94%, keeping at-risk individuals vaccinated could prove beneficial to them and help reduce resource demands associated with the management of severe SARS-CoV-2 infection.^7^ Of importance, although individuals experiencing breakthrough infections during the Delta surge had lower antibody levels, this was not the case during the Omicron surge. Lower antibody levels observed in Delta-era infections may also have resulted as a time-artifact because booster dose recommendations were initiated after onset of the Delta surge.

Most SARS-CoV-2 vaccine breakthrough infections reported in this study occurred during the Delta and Omicron surges in West Virginia. Of interest, approximately half of the study participants with breakthrough infections reported between October 2021 and January 2022 had received a booster dose before infection. Although individuals with lower IgG levels were more likely to experience an infection during the Delta surge, no significant difference was observed in antibody indexes between individuals with vaccine breakthrough infections during the Omicron-era surge compared with those without infection. Previously published reports^11,12,13^ have shown decreased vaccine effectiveness against infections from Delta and, more prominently, Omicron variants, suggesting that serologic correlates of protection are likely to be SARS-CoV-2 variant specific. This finding supports the idea that increasing SARS-CoV-2 immunity in the human population will accelerate viral evolution.^14^ Moreover, given demonstrated attenuation of existing vaccines to evolving variants, variant-specific versions of SARS-CoV-2 vaccines are under development.^15^ Although our substudy of 95 participants indicated, as have other studies,^16,17^ a correlation of neutralizing antibody to levels of IgG RBD, these studies were conducted using the Wuhan strain and may not be generalizable to variants such as Delta and Omicron.

### Limitations

Limitations of this study include single-point serologic measurement. In addition, many infections reported in this study occurred 2 to 3 months after specimen collection, and the observed antibody levels likely do not precisely reflect those at the time infection occurred. Nonetheless, it is assumed that subsequent antibody levels were not higher unless breakthrough infections or vaccine booster occurred. In addition, illness severity was not measured as part of the study design, and the correlation of symptom severity with antibody level remains unknown. Generalization is limited because the study population was mostly composed of White women. Finally, past infection, vaccination, and booster status were determined from reporting by the nursing homes as well as by state databases, and accuracy of these data sources is unknown.

## Conclusions

Overall, the findings of this cross-sectional study showed a decrease in anti-RBD IgG levels after vaccination and infection; however, data presented in this study are not conclusive in providing a serologic correlate of protection against SARS-CoV-2 infection. The findings of this cross-sectional study suggest that among nursing home residents, COVID-19 vaccine boosters are important and updated vaccines effective against emerging SARS-CoV-2 variants are needed.

## Notes

Supplement. **eFigure 1.** Distribution of Anti-RBD IgG Indices Among Participants With Reported Infection History (Red) and Without Infection History (Blue) **eFigure 2.** Correlation of Anti-RBD IgG Indices and Log ECL Signal Obtained From ACE-2 Binding Inhibition Assay
