# Supplementary material for: Persistence and Protective Potential of SARS-CoV-2 Antibody Levels After COVID-19 Vaccination in a West Virginia Nursing Home Cohort
Source: JAMA Netw Open. 2022 Sep 13;5(9):e2231334. doi: 10.1001/jamanetworkopen.2022.31334 (PMC9471977; doi:10.1001/jamanetworkopen.2022.31334)
Supplement: Supplement. — eFigure 1. Distribution of Anti-RBD IgG Indices Among Participants With Reported Infection History (Red) and Without Infection History (Blue) eFigure 2. Correlation of Anti-RBD IgG Indices and Log ECL Signal Obtained From ACE-2 Binding Inhibition Assay [file jamanetwopen-e2231334-s001.pdf]

Supplementary Online Content

Smoot K, Yang J, Tacker DH, et al. Persistence and protective potential of SARS-CoV-2 antibody levels after COVID-19 vaccination in a West Virginia nursing home cohort. *JAMA Netw Open*. 2022;5(9):e2231334. doi:10.1001/jamanetworkopen.2022.31334

eFigure 1. Distribution of Anti-RBD IgG Indices Among Participants With Reported Infection History (Red) and Without Infection History (Blue)

eFigure 2. Correlation of Anti-RBD IgG Indices and Log ECL Signal Obtained From ACE-2 Binding Inhibition Assay

This supplementary material has been provided by the authors to give readers additional information about their work.

© 2022 Smoot K et al. *JAMA Network Open.*

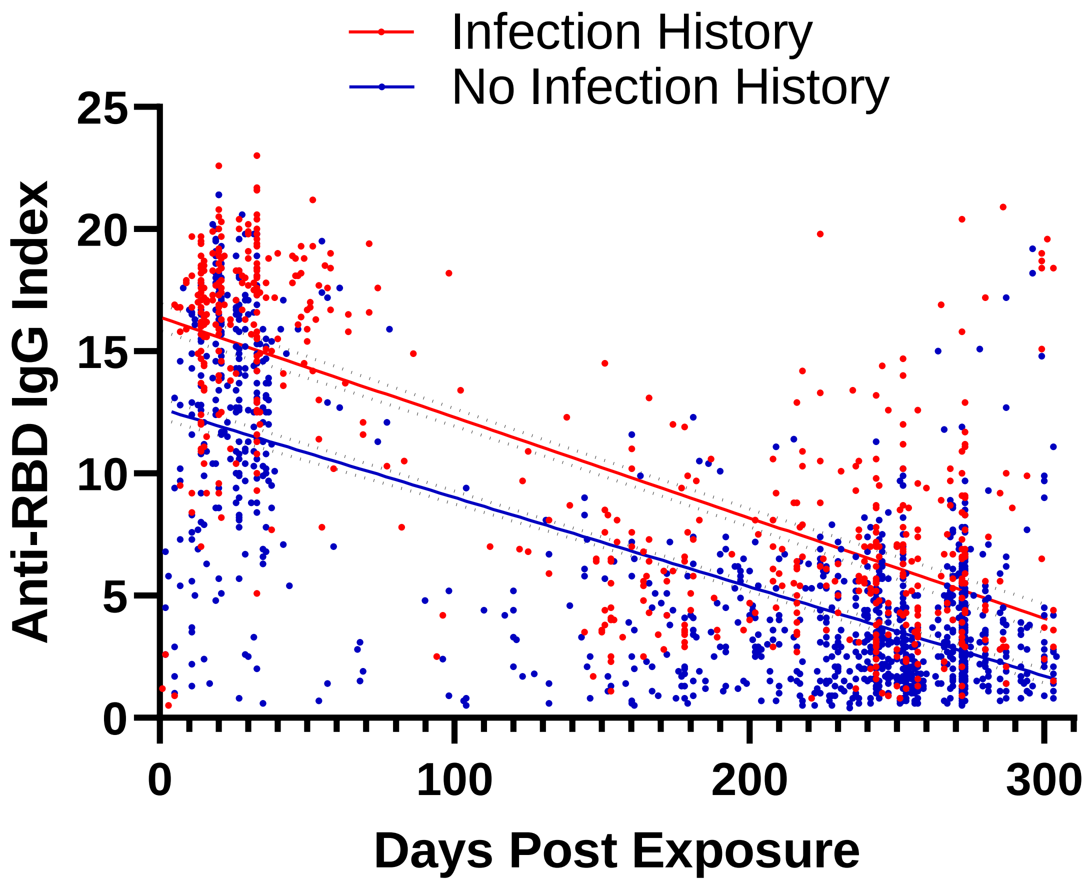

eFigure 1. Distribution of Anti-RBD IgG Indices Among Participants With Reported Infection History (Red) and Without Infection History (Blue)

Exposure is defined as an immune event (I.e., vaccination or infection).

© 2022 Smoot K et al. *JAMA Network Open.*

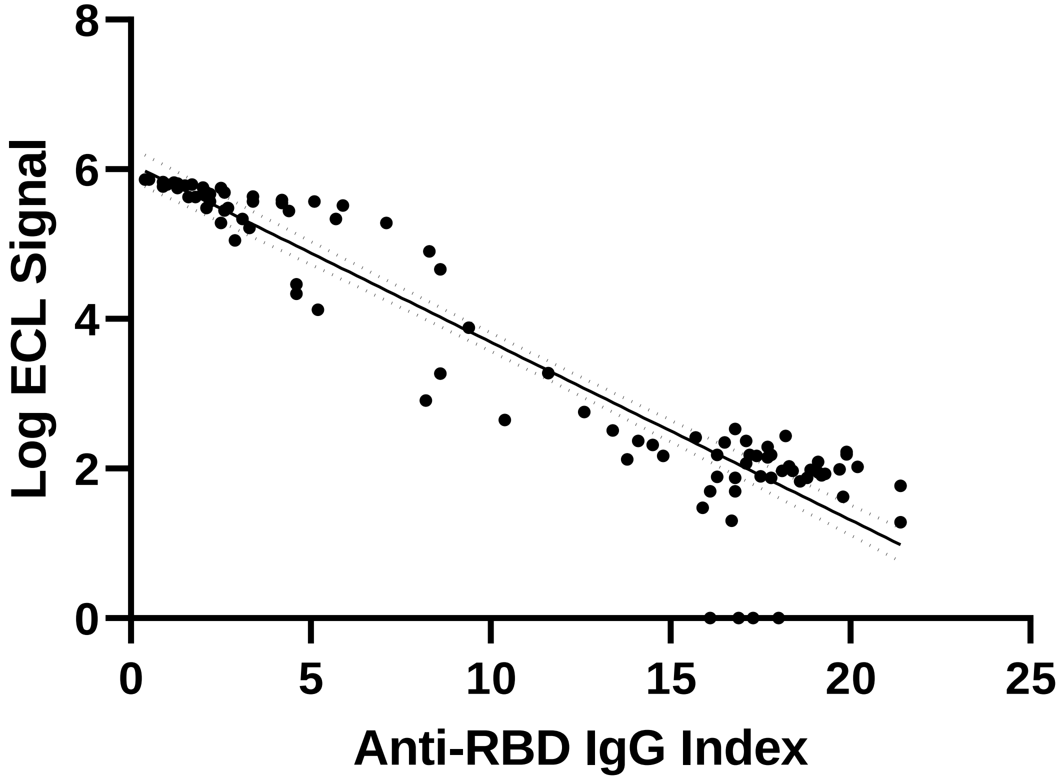

eFigure 2. Correlation of Anti-RBD IgG Indices and Log ECL Signal Obtained From ACE-2 Binding Inhibition Assay

© 2022 Smoot K et al. *JAMA Network Open.*
